# A study on the flexibility of enzyme active sites

**DOI:** 10.1186/1471-2105-12-S1-S32

**Published:** 2011-02-15

**Authors:** Yi-Zhong Weng, Darby Tien-Hao Chang, Yu-Feng Huang, Chih-Wei Lin

**Affiliations:** 1 Department of Computer Science and Information Engineering, National Taiwan University, Taipei, 106, Taiwan; 2 Department of Electrical Engineering, National Cheng Kung University, Tainan 701, Taiwan

## Abstract

**Background:**

A common assumption about enzyme active sites is that their structures are highly conserved to specifically distinguish between closely similar compounds. However, with the discovery of distinct enzymes with similar reaction chemistries, more and more studies discussing the structural flexibility of the active site have been conducted.

**Results:**

Most of the existing works on the flexibility of active sites focuses on a set of pre-selected active sites that were already known to be flexible. This study, on the other hand, proposes an analysis framework composed of a new data collecting strategy, a local structure alignment tool and several physicochemical measures derived from the alignments. The method proposed to identify flexible active sites is highly automated and robust so that more extensive studies will be feasible in the future. The experimental results show the proposed method is (a) consistent with previous works based on manually identified flexible active sites and (b) capable of identifying potentially new flexible active sites.

**Conclusions:**

This proposed analysis framework and the former analyses on flexibility have their own advantages and disadvantage, depending on the cause of the flexibility. In this regard, this study proposes an alternative that complements previous studies and helps to construct a more comprehensive view of the flexibility of enzyme active sites.

## Background

Enzymes are organic catalysts that play an important role in various biological processes. It has been shown that the speeds of enzymatic reactions are much faster than non-enzymatic ones [1]. Such catalytic processes do not occur at arbitrary regions but at specific sites—usually one or at most a few—of an enzyme. The sites of catalysis have been called “active sites”. For rapidly and specifically distinguishing between closely similar compounds, the physiochemical properties of active sites are expected to be highly conserved. A good example is the Ser-His-Asp catalytic triad of serine proteases, where the relative positioning of these three catalytic residues remains rigid in enzymes with very different global structures [2].

However, this expectation of invariability is not applicable to all active sites. Several studies have found homologous enzymes that can perform different catalyses via different mechanisms [3-5]. Grishin has demonstrated examples of achieving similar reaction chemistries in completely different ways [6]. Such conditions often involve structural changes in the proximity of the active sites [6,7]. The variability of structural or chemical characteristics among binding sites has also been discussed as “flexibility” [8], “plasticity” [9] and “stability” [10], where stability is opposite to variability. We focus on the structure rather than sequence variations and adopt “flexibility” in this manuscript.

In 2002, Todd *et al.* reported 17 examples of enzyme homologues having obvious structural changes within the active sites [9]. They did not perform any computational analysis to measure the flexibility of the collected active sites. Instead, Todd and colleagues provided valuable discussion on this issue based on many examples. They confirmed the existence of flexible active sites and proposed several evolutionary possibilities that could result in the flexibility. Another study conducted by Kahraman *et al.* analyzed the binding sites associated with the same ligand [11]. Kahraman and colleagues pre-selected nine ligand types of different sizes and flexibilities and analyzed 100 Protein Data Bank (PDB) [12] structures of binding sites that bind the pre-selected ligands. Their work used spherical harmonics as the shape descriptor to model the binding sites and invoked a shape comparison algorithm to quantify the flexibility of binding sites [13]. They have shown that shape variations between a binding site and its ligand counterpart are correlated. In 2009, Saranya and Selvaraj systematically analyzed the variation of protein binding sites of 200 PDB structures collected from eight protein families [14]. They focused on the cavity volume of protein binding sites and observed variations of the cavity volume of the same site when binding different ligands. Saranya and Selvaraj concluded that the volume of both the binding site and its ligand counterpart are highly correlated to the atom-atom interactions in the binding site.

The above three studies share a basic analysis philosophy: identifying some active sites known to be flexible and then investigating their flexibilities. Furthermore, a common assumption that the flexibility of active sites comes from compensating the ligand flexibilities led previous studies to associate site flexibility with the ligand counterpart. This assumption largely reduces the data—requiring protein-ligand complexes—available for analysis as well as the potential to understand new classes of flexible sites.

In this regard, this study proposes an analysis framework aimed at identifying flexible active sites rather than analyzing known flexible ones. We compile 58 groups of annotated active sites from the CSA (Catalytic Site Atlas) database [15], which is the largest catalogue of catalytic residues. This dataset of active site groups contain 1,612 PDB structures, spanning 46 EC (Enzyme Commission) codes and 188 protein families. In this study, the flexibility of an active site group is obtained from the pair-wise structure alignments among that group. Here we adopt the CLoSA (Constraint-based Local Structure Alignment) algorithm [16] as the alignment tool; this is designed for and has been shown to be successful in discriminating active sites. The proposed method requires less manual intervention and is suitable for analyzing larger dataset than existing analysis methods. Our experimental results show that it (a) gives results consistent with the previous works based on manually identified flexible active sites and (b) is capable of identifying potentially new flexible active sites.

To summarize, this study proposes a new strategy of data preparation and a corresponding analysis framework. The collected dataset is constructed using automatically extracted geometrical templates without manual intervention. This dataset is relatively large in comparison with those used in the previous studies on active site flexibility. This alleviates the bias of individual differences in protein structures due to unfavorable factors such as crystallization. In addition, the collected dataset can be used to analyze conformational changes during the catalytic reaction and/or between unbound and bound forms. This is an advantage that it can detect various types of flexibility caused by the factors different to known factors such as flexible ligands. However, this is also a disadvantage that further filtering is required if specefic types of flexiblity are of interest. Some limitations of the proposed analysis framework are discussed at the end of the “Results and Discussion” section. Namely, different data collection strategies may observe distinct causes of flexibility. In this regard, this study proposes an alternative analysis complementing previous studies and helps to construct a more comprehensive view of the flexibility of enzyme active sites.

## Results and discussion

This section first describes the dataset, including how the data is prepared and how the physicochemical properties of active sites are obtained using local structure alignment. Next, the measured flexibilities of active sites and their relation to the corresponding ligands are present. At the end of this section, we outline some case studies and discuss the limitations of the proposed analysis framework.

### Dataset

We started by collecting data from the CSA database, the largest database of active site annotations. This data preparation method has several advantages and provides analyses alternative to previous studies starting from ligands. First, the collected structures are not required to be complexes. The method can be used to analyze the flexibility of unbound structures. Second, CSA entries contain annotated catalytic residues, which can be used to “calibrate” the alignment. Because active sites are small and we focus on flexible ones, there are usually multiple alignments of two site structures with very different aligned residues. How to choose the correct alignment largely affects the measured flexibility. In this study, alignments aligning catalytic residues better are first considered.

CSA version 2.2.11 contains 91,840 entries, spanning 1,208 EC codes and 3,325 protein families. Each CSA entry is composed of two to six residues of a PDB structure. All the 91,840 CSA entries distribute in 23,449 PDB structures. To simplify the analysis, entries where the corresponding PDB structure has multiple EC codes are excluded. This study focuses on oxidoreductases, as their the application in performing synthetic transformations is an important area [17]. The remaining set comprises 5,613 CSA entries, where 324 entries are obtained from the literature and the other 5,289 entries are homologues of the 324 literature entries. Some of the 324 literature entries overlap, that is, they share some residues in the same PDB structure. In the proposed analysis framework, the overlapping literature entries and their homologous entries are considered as a group. Accordingly, the 5,613 CSA entries are clustered into 151 active site groups.

Then pair-wise structure alignments are performed on each of the 151 active site group using the CLoSA algorithm [16]. Each alignment includes information of transformations to superimpose the two aligned local structures and a list of matched residues. More details of the structure alignment can be found in the “Methods” section. Some active site groups are further removed during this step. First, small groups with less than ten PDB structures are discarded. Second, a group is considered as too diverse if CLoSA fails 25% pair-wise alignments or the proportion of residues successfully aligned is less than 50% in that group. This diversity could be due to the existence of subgroups of the active site, which will mislead the flexibility analysis. The final dataset of this study is a collection of 58 active site groups from 1,612 PDB structures across 46 EC codes and 188 protein families.

### Measuring the flexibility of enzyme active sites

Based on the results of pair-wise alignment, this study provides several physicochemical properties of local structures for measuring the flexibility (Table 1). The size of an active site is defined as the average number of residues of the associated local structures. #align and %align measure the quantity of matched residues, where #align is the number of residues successfully aligned in the pair-wise alignment and %align is the ratio of #align to the size number of residues of the smaller local structure in the alignment. The stdev (standard deviation) of %align is used as the flexibility index in this study. This index, as will be elaborated in the following subsections, is consistent with previous works on flexibility. Charge in Table 1 represents the electrostatic state around the active site by averaging the charge of each associated local structure of an active site group. Here the charge of a local structure is the sum of its amino acid charges (1:positive, 0:neutral and -1:negative) according to Klein *et al*.’s study [18]. The calculation of Polar and ASA (accessible surface area) in Table 1 is similar to Charge, except that the per amino acid polarity is obtained from the study of Radzicka and Wolfenden [19] and the “per amino acid ASA” is calculated with the DSSP (Dictionary of Protein Secondary Structure) package [20].

**Table 1:** Physicochemical properties of an active site group

Property	Description
Size	number of residues
#align	number of matched residues in the alignment
%align	ratio of residues successfully matched in the alignment (%)
RMSD	root mean square deviation of the matched residues (Å)
Charge	sum of the electrostatic preference of all residues
Polarity	sum of the polar preference of all residues
ASA	sum of accessible surface area of all residues (Å^2^)

### This proposed flexibility index is consistent with previous works

All information, including the physicochemical properties of the active site and some statistics such as number of CSA and PDB entries, of the 58 active site groups can be found in Additional file 1. Table 2 shows the ten most flexible active sites and the ten most rigid groups identified using the proposed method. In the ten most flexible groups, 1powA, 1getA and 1d4cA bind FAD (flavin adenine dinucleotide); while 4mdhA, 1arzA and 1emdA bind NAD (nicotinamide adenine dinucleotide). In the flexibility study of Kahraman *et al*. [11], FAD and NAD were selected as the biggest and most flexible molecules. 1dhfA binds NDP (nicotinamide adenine dinucleotide phosphate), which is simply NAD with a third phosphate group attached and has very similar chemistry of that of NAD [21,22]. In the ten most rigid groups, 1dveA, 3nosA, 1dj1A, 7atjA and 2cpoA bind heme; 1idtA and 1fcbA bind FMN (flavin mononucleotide); while 1aopA binds SRM (siroheme), a heme-like chromophore with a closely similar prosthetic group to heme [23,24]. Heme and FMN were reported to be slightly flexible in Kahraman *et al*.’s study.

**Table 2:** The most flexible/rigid active sites identified with the proposed method

Active site^1^	EC code	#LC^2^	Size	#align	%align^3^	RMSD	Charge	Polarity	ASA
The ten most flexible active sites									
1powA	1.2.3.3	22	11±2.4	7.7±3.1	68±28	0.5±0.5	-0.8±1.1	-1.0±0.8	441.9±70.3
1n2cB	1.18.6.1	77	7±0.7	5.0±1.5	73±23	0.9±1.0	-1.0±0.2	3.3±0.6	102.5±52.4
4mdhA	1.1.1.37	36	8±1.7	5.4±1.6	68±22	0.3±0.3	-1.0±0.0	0.8±0.7	98.8±37.3
1arzA	1.3.1.26	28	9±1.0	6.4±2.0	69±21	0.6±0.8	-0.1±0.7	1.7±1.5	458.6±105.1
1dhfA	1.5.1.3	91	9±2.1	4.9±1.6	56±21	0.9±0.7	-1.5±0.9	1.2±1.8	412.3±88.4
1getA	1.8.1.7	35	17±5.7	11.7±5.1	73±20	0.3±0.2	0.3±1.0	5.8±1.9	380.2±104.4
1emdA	1.1.1.37	111	9±2.0	5.4±1.6	61±20	1.0±0.8	-1.7±0.8	-0.2±1.1	105.1±41.5
1d4cA	1.3.99.1	45	18±2.0	11.7±3.4	64±20	1.2±0.7	0.6±0.6	-1.1±1.1	289.4±124.8
1a05A	1.1.1.85	53	12±4.3	7.0±2.9	61±19	0.4±0.4	-1.2±0.7	0.8±1.1	336.0±94.3
1n2cE	1.18.6.1	45	7±1.2	6.0±0.5	88±18	1.2±0.7	0.4±0.7	-2.4±0.9	124.8±53.3
The ten most rigid active sites									
1dveA	1.14.99.3	76	25±2.1	20.3±2.8	85±8	0.7±0.2	-1.7±0.6	2.7±2.3	599.0±125.5
3nosA	1.14.13.39	264	20±1.7	17.7±2.3	93±8	0.3±0.2	0.8±0.7	3.1±0.8	504.2±82.0
1dj1A	1.11.1.5	68	15±2.7	12.3±1.7	88±8	0.2±0.1	0.1±0.5	2.7±1.5	301.6±55.0
1idtA	1.5.1.34	28	9±0.4	8.1±0.8	94±7	0.2±0.1	2.3±0.5	-3.3±0.8	286.2±68.3
7atjA	1.11.1.7	39	14±1.1	12.6±1.0	95±6	0.2±0.1	-1.0±0.0	0.4±0.5	280.4±58.8
1n2cA	1.18.6.1	41	17±1.6	15.2±1.2	93±5	0.2±0.1	2.0±0.0	-1.3±0.8	255.5±32.0
1fcbA	1.1.2.3	20	19±2.8	16.6±1.6	94±5	0.3±0.1	0.7±0.8	-0.4±0.9	281.2±63.6
1dodA	1.14.13.2	35	11±0.9	9.9±0.8	98±4	0.3±0.2	-0.4±0.5	2.3±0.8	66.7±11.3
2cpoA	1.11.1.10	12	10±0.8	9.8±0.7	98±4	0.2±0.3	-3.0±0.0	-0.7±0.5	104.8±6.2
1aopA	1.8.1.2	11	21±0.9	20.1±1.1	98±2	0.3±0.1	4.9±0.3	-4.5±0.7	533.8±44.5

^1^Each of the 20 active site groups includes overlapping CSA literature entries and a number of homologous entries identified with those literature entries. Here we use the literature entry to represent an active site group. ^2^Number of local structures associated with the active site group. ^3^The 20 active site groups are sorted by the standard deviation of %align, which is the flexibility index of this study.

As a result, 15 of the 20 groups bind the ligands discussed in previous flexibility studies [11,25], where the flexible and rigid sites/ligands are manually selected. These results reveal, in addition to the good performance of the proposed analysis framework, that the flexibility of protein-ligand binding sites can be observed even with distinct data preparation strategies.

In Table 2, RMSD has the highest correlation to the flexibility index. However, the correlation (*R*^2^=0.35) is low, because RMSD relies heavily on the number of items under consideration. If two corresponding residues in two local structures are too distant to be aligned, a large conformational change is indicated. However, in the RMSD calculation, this residue pair will be discarded and usually lead to a smaller RMSD. Thus, a rigid active site must have a small RMSD (see the ten rigid groups in Table 2), but a small RMSD does not guarantee a rigid active site (see the ten flexible groups in Table 2). In this regard, the stdev of RMSD is more suitable than RMSD for measuring how the alignments vary among a group. The higher correlation (*R*^2^=0.55) of the stdev of RMSD to the flexibility index concurs with this argument.

In addition to geometric properties, this study also analyzes the charge, polarity and surface area of active sites. In Table 2, the charge of all flexible active sites is in the range of [-2,2] and is either slightly negative or nearly neutral. Conversely, four rigid active sites (1idtA, 1n2cA, 2cpoA and 1apoA) have a larger charge. However, though Charge is more highly correlated to the flexibility index than Polarity and ASA, the *R*^2^ (0.14 for Charge and 0.22 for the stdev of Charge) is still limited. This suggests that the chemical characteristics near the active sites are much more conserved than or irrelevant to the geometric characteristics. Otherwise, more chemical properties should be considered. The last observation in Table 2 is that ASA is completely uncorrelated to the flexibility index (*R*^2^=0.00), but its stdev has a comparable correlation (*R*^2^=0.15) to Charge. A reasonable explanation is the partially bound ligands, which sink only partially to an active site with the other end protruding into the solvent [11]. Such conditions make ASA a less useful measure. However, its stdev can slightly detect the surface variation of the bound end of the ligand.

### Flexibility of the ligand counterpart

This subsection verifies the correlation of the flexibility of the active site to its ligand counterpart. To identify the ligand counterpart of an active site, the closest hetero molecule is considered. This study associates an active site with a set of the PDB structures, thus the closest hetero molecules could vary owing to the absence of some ligands in different PDB structures. We guarantee that the selected ligand for each active site (a) is the most frequent hetero molecule observed in the associated PDB structures and (b) has at least one heavy atom whose distance to a heavy atom of the active site is closer than 6.5Å. The selected ligands of 19 active sites in Table 2 satisfy the above conditions. The only exception is 1a05A of which the most frequent hetero molecule is the SO4 (sulfate ion), but SO4 only appears five times in the 25 associated PDB structures and three of them are distant (>6.5Å) from the active site.

The counterparts of the 20 active sites cover ten ligands (Table 3). Table 4 shows the nine properties of ligands adopted in this study, where eight properties, except Dist., are obtained by querying the PubChem database [26]. Note that identical ligands could have different properties depending on the complexes they belong to. The small differences come from the number of hydrogen atoms that are dissociated in the solvent. For example, the HEMs associated with 1dveA and 3nosA have molecular formulas “C_34_H_32_FeN_4_O_4_” and “C_34_H_36_FeN_4_O_4_”, differing in four hydrogen atoms. The representative PDB structure shown in the first column of Table 3 is used to query the PubChem database.

**Table 3:** The ligand counterparts of the identified active sites

Active site	Ligand	Formula	#atom	MW	Dist.	Charge	#HD	#HA	#rotatable	TPSA
The ten most flexible active sites										
1powA	FAD^1^	C_27_H_34_N_9_O_15_P_2_^+^	53	786.6	3.1	1	10	20	13	357.0
1n2cB	CLF^2^	Fe_8_H_7_S_7_^-7^	15	678.3	2.4	-7	0	7	0	0.0
4mdhA	NAD^3^	C_21_H_28_N_7_O_14_P_2_^+^	44	664.1	4.3	1	8	18	11	318.0
1arzA	NAD	C_21_H_28_N_7_O_14_P_2_^+^	44	664.1	3.4	1	8	18	11	318.0
1dhfA	NDP^4^	C_21_H_29_N_7_O_17_P_3_^+^	48	744.4	3.7	1	9	21	13	365.0
1getA	FAD	C_27_H_34_N_9_O_15_P_2_^+^	53	786.6	2.8	1	10	20	13	357.0
1emdA	NAD	C_21_H_28_N_7_O_14_P_2_^+^	44	664.1	3.4	1	8	18	11	318.0
1d4cA	FAD	C_27_H_34_N_9_O_15_P_2_^+^	53	786.6	3.4	1	10	20	13	357.0
1a05A^5^	–	–	–	–	–	–	–	–	–	–
1n2cE	ADP^6^	C_10_H_15_N_5_O_10_P_2_	27	427.2	3.5	0	6	14	6	233.0
The ten most rigid active sites										
1dveA	HEM^7^	C_34_H_32_FeN_4_O_4_	43	616.5	2.1	0	2	8	8	101.0
3nosA	HEM	C_34_H_32_FeN_4_O_4_	43	620.5	2.3	0	2	8	8	101.0
1dj1A	HEM	C_34_H_32_FeN_4_O_4_	43	620.5	3.4	0	2	8	8	101.0
1idtA	FMN^8^	C_17_H_22_N_4_O_9_P^+^	31	457.4	2.7	1	7	10	7	202.0
7atjA	HEM	C_34_H_32_FeN_4_O_4_	43	616.5	3.4	0	2	8	8	101.0
1n2cA	HCA^9^	C_7_H_10_O_7_	14	206.2	3.4	0	4	7	6	132.0
1fcbA	FMN	C_17_H_22_N_4_O_9_P^+^	31	457.4	3.2	1	7	10	7	202.0
1dodA	PHB^10^	C_7_H_6_O_3_	10	138.1	2.8	0	2	3	1	57.5
2cpoA	HEM	C_34_H_32_FeN_4_O_4_	43	620.5	2.8	0	2	8	8	101.0
1aopA	SRM^11^	C_42_H_46_FeN_4_O_16_	63	918.7	2.5	0	8	20	20	302.0

The details of each column are described in Table 4. ^1^flavin adenine dinucleotide. ^2^Fe(8)-S(7) cluster. ^3^nicotinamide adenine dinucleotide. ^4^nicotinamide adenine dinucleotide phosphate. ^5^No appropriate ligand is available for this active site group. ^6^adenosine triphosphate. ^7^heme. ^8^flavin mononucleotide. ^9^3-hydroxy-3-carboxy-adipic acid. ^10^4-hydroxybenzoic acid. ^11^siroheme.

**Table 4:** Physicochemical properties of a ligand

Property	Description
Formula	chemical formula of the compound
#atom	number of heavy atoms
MW	molecular weight; each element is weighted for its natural isotopic abundance
Dist.	average distance of all heavy atoms of the active site to those of the ligand
Charge	formal charge of the compound
#HD	number of hydrogen bond donors in the compound
#HA	number of hydrogen bond acceptors in the compound
#rotatable	number of rotatable bonds
TPSA	topological polar surface area; surface area estimated as polar (Å^2^)

**Figure 1:**
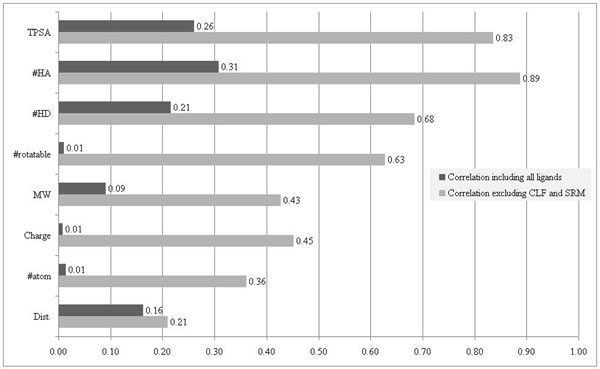
Correlation between the flexibilities of an active site and its ligand counterpart.

In Table 3, the property most correlated to the flexibility index is #HA. However, the correlation is limited (*R*^2^=0.31). The next most correlated properties are TPSA (*R*^2^=0.26), #HD (*R*^2^=0.21) and Dist. (*R*^2^=0.16). All the remaining properties are uncorrelated to the flexibility index (*R*^2^<0.1). When further checking the #HA, we observe that all ligands associated with flexible sites have more H-bond acceptors except CLF, the only inorganic compound. The Fe atom may make the binding mechanism distinct to other ligands. On the other hand, all ligands associated with rigid sites have fewer H-bond acceptors except SRM. This is an outlier of the ten rigid sites in terms of MW, #HD, #rotatable and #atom. Thus, we exclude these two ligands. The correlations of all of the properties are significantly increased (Figure 1).

After excluding CLF and SRM, #HA, TPSA and #HD are still the most correlated properties with higher *R^2^* of 0.89, 0.83 and 0.68, respectively. The next most correlated property becomes #rotatable. It has a much better *R*^2^ of 0.63 than Dist. (0.43), the property more correlated to the flexibility index in the analysis without excluding CLF and SRM. In principle, #rotatable is a good indicator of the flexibility of a ligand, while Dist. might be misled by a few atoms. This suggests that the analysis that excludes CLF and SRM is more reasonable.

According to the results of this study, the flexibility of an active site is correlated to both the topological polar surface area and number of H-bond acceptors of its ligand counterpart, followed by the number of H-bond donors and of rotatable bonds. These four properties, however, are not universal to all kinds of ligands. More efforts are required to understand the flexibility of active sites binding to special ligands, such as inorganic compounds.

### Case studies

This subsection uses three examples identified by the proposed method to discuss the active site flexibility. The first example is an active site which binds FAD, where a disordered segment is observed enabling adaptation to the flexible ligand. The second example is a dephosphorylation reaction, where the active site varies according to the chemical changes between the reactant and the product. The third example is a zinc site of two geometric forms. The last two examples demonstrate how the proposed method detects flexible active sites without known flexible ligand counterparts. These results suggest that the proposed framework helps to identify novel flexible active sites as well as novel flexibility types worthy for furthey studies.

**Figure 2:**
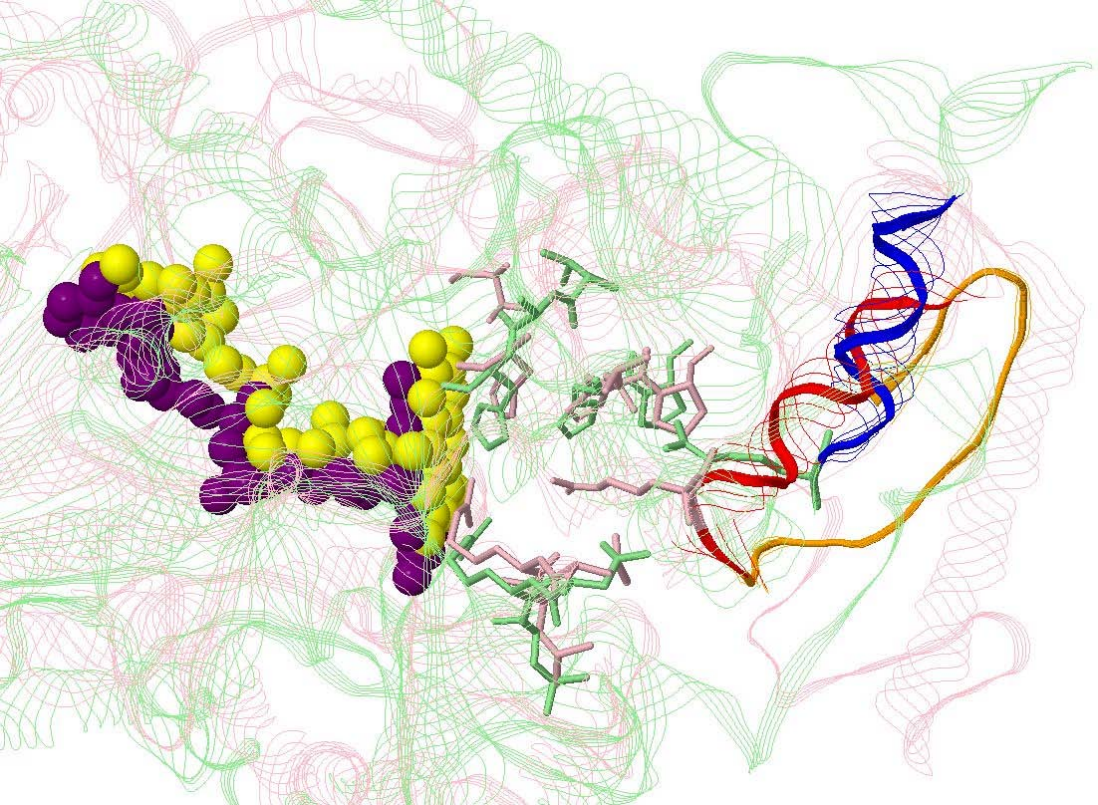
**Alignment between 2b7sA and 3cirA, two structures associated with the same active site of succinate dehydrogenase (EC code: 1.3.99.1).** The proteins are represented as strands and the active sites as. 2b7sA is pink and 3cirA is green. The corresponding ligands are represented as balls (yellow for 2b7sA and purple for 3cirA). The mismatched helices are red (T401–A411 of 2b7sA) and blud (R287–H296 of 3cirA). The segment in 2b7sA (L388–D400) corresponding to the disordered segment in 3cirA (L254–P286) is orange.

The first example is the alignment between 2b7sA and 3cirA, both belonging to the 1d4cA active site group. The enzyme of this active site group is succinate dehydrogenase (EC code: 1.3.99.1) of which the corresponding ligand is FAD. In this case, nine of 16 residues in the local structures and three of four catalytic residues are successfully matched (Figure 2). The only unmatched catalytic residue (R402 of 2b7sA and R287 of 3cirA) locates on a helix (T401–A411 of 2b7sA and R287–H296 of 3cirA). This helix is denoted as *h*. We performed global structure alignment on 2b7sA and 3cirA and found that there is an obvious movement of *h*. We further checked the proximity of *h* and identify a disordered segment (L254–P286), denoted as *d*, in 3cirA. However, the corresponding segment of *d* in 2b7sA (L388–D400) is ordered. In the binding process of this active site, the helix *h* plays the role of latch to fix the ligand after it enters the active site. The disordered segment *d* is used to fasten the latch. As a result, the ordered and disordered forms of *d* reveal that 2b7sA and 3cirA could represent two states of the same binding process, leading to the observed flexibility.

The second example is the alignment between 2c8vA and 1nipB, both belonging to the 1n2cE active site group. The enzyme of this active site group is nitrogenase (EC code: 1.18.6.1). There are two corresponding ligands for this active site, ATP (adenosine triphosphate) and ADP (adenosine diphosphate). This is a typical dephosphorylation reaction where ATP is a coenzyme transporting chemical energy and will be converted into its precursor, ADP, after the reaction:

8 reduced ferredoxin + 8 H^+^ + N_2_ + 16 ATP + 16 H_2_O = 8 oxidized ferredoxin + H_2_ + 2 NH_3_ + 16 ADP + 16 phosphate

**Figure 3:**
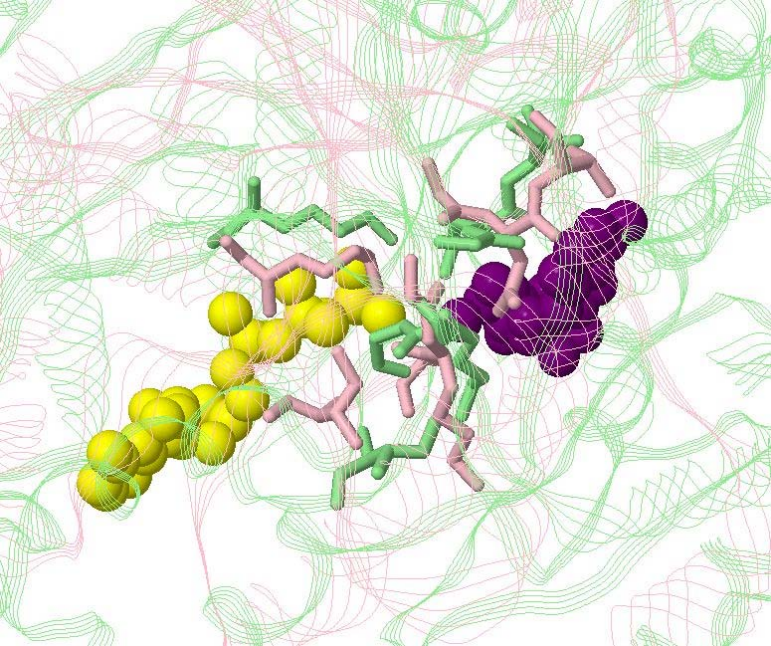
**Alignment between 2c8vA and 1nipB, two structures associated with the same active site of nitrogenase (EC code: 1.18.6.1).** The proteins are represented as strands and the active sites as sticks. 2c8vA is pink and 1nipB is green. The corresponding ligands are represented as yellow (ATP of 2c8vA) and purple (ADP of 1nipB) balls.

Thus, in this case, although ATP and ADP are different ligands, they should be regarded as the starting and ending states of the same compound in this reaction. Figure 3 shows the alignment. Unlike the previous example, there is no specific residue that has obvious movement in the local structures. Most of the residues are successfully matched in this alignment, but the RMSD of 3.1Å is large. Figure 3 clearly reveals that both ATP and ADP attach the active site, but they do not overlap at all. This suggests that the ATP/ADP compound “shifts” along the active site during the reaction, leading to the observed flexibility.

**Figure 4:**
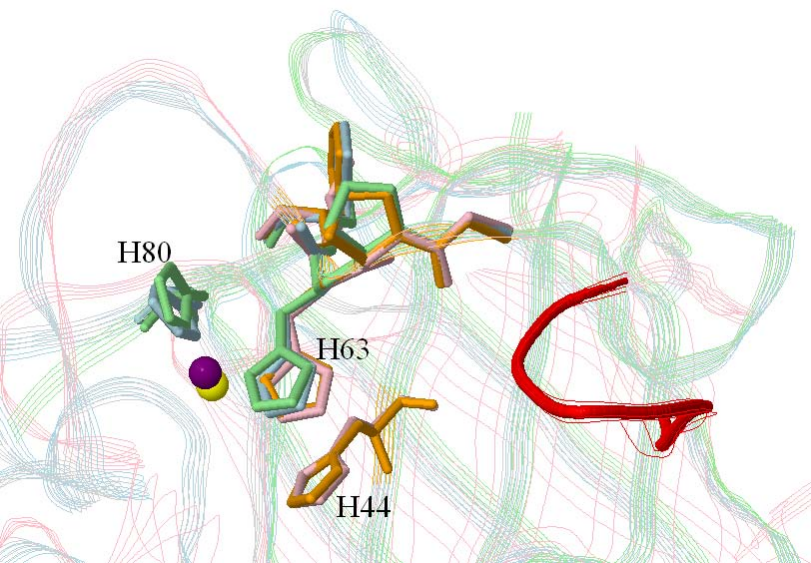
**Alignment among 1esoA, 1oezZ, 1e9qB and 1uxlB, four structures associated with the same active site of Cu,Zn superoxide dismutase (EC code 1.15.1.1).** The proteins are represented as strands and the active sites as sticks. 1esoA is pink, 1oezZ is green, 1e9qB is orange and 1uxlB is blue. The corresponding zinc ions are represented as balls (yellow for 1esoA and purple for 1eozZ). The seven-residue insertion of 1esoA (K54^A-A54^G) is red. The caret symbol (^) indicates an insertion code in PDB format, which reveals that these seven residues were discovered later after the first submission of 1esoA to PDB. All four structures share a histidine (H63 of 1esoA). The second histidine (H44 of 1esoA) only appears in 1esoA and 1e9qB, while the third histine (H80 of 1oezZ) only appears in 1oezZ and 1uxlB.

The third example is the zinc site of Cu,Zn superoxide dismutase (SOD). Analyzing the flexibility from its ligand counterpart, the zinc ion, is not applicable. Thus, we manually looked into the results of pair-wise structure alignments and speculated that there are two subgroups in this active site group. The structures in the same subgroup match well but those in different subgroups match badly. Here we use the alignment among 1esoA, 1e9qB, 1oezZ and 1uxlB to demonstrate the observed grouping phenomenon (Figure 4). All of them belong to the 2jcwA active site group (EC code: 1.15.1.1). The four structures form two sets, 1esoA-1e9qB and 1oezZ-1uxlB. In Figure 4, the residues connected to H63 in all the four local structures matched well. It has been shown that the H63 histidine does coordinate to the zinc ion [27]. The unmatched residues were H44 (only appears in 1esoA and 1e9qB, the first subgroup) and H80 (only appears in the second subgroup). According to our survey the H44 histidine has been speculated to be more related to the copper ion of Cu,Zn [28,29]; while the H80 histidine has not been mentioned in any surveyed Cu,Zn SOD studies. This observation of the flexibility of Cu,Zn SOD concurs with the results of two previous studies based on laboratory experiments. Bauer *et al*. applied the PAC (perturbed angular correlation) method [27] to study of the zinc site of Cu,Zn SOD and observed two different, pH independent, PAC spectrums. They concluded that Cu,ZN SOD has at least two geometric forms for the zinc site. Another study by Falconi *et al*. [30] analyzed the prokaryotic and eukaryotic Cu,Zn SODs with limited proteolysis and molecular dynamics simulation. They confirmed that a seven-residue insertion of the *Escherichia coli* Cu,Zn SOD forms an alternative organization of the active site, compared to those eukaryotic ones. The seven-residue insertion is observed in our first set (K54^A–A54^G of 1esoA) but not the second set. This further confirms our speculated bi-forms of Cu,ZN SOD is probably the same as the one observed in [30].

### Limitations and problems

This study proposes an alternative analysis framework of collecting data without ligand information and summarizing flexibility by properties based on atomic coordinate comparison. The above results and discussion focus on the advantages of the proposed method, however, it suffers from some limitations. The most challenging problem is the existence of subgroups of an active site. This can result from active sites that have multiple binding forms or those that can bind multiple distinct ligands. The third example in the previous subsection reveals the potential of detecting subgroups of the proposed method. However, the analysis is still manual. Active site clustering, which itself is an important issue, is required to tackle this problem.

The second problem is the limitation of the selected physicochemical properties. As shown in the case studies, the flexibility of a chemical compound is a complicated process and is difficult to describe by some measures. A reasonable solution to this problem is to design different measures for distinct binding conditions. The CLF ligand in Table 3 is a good example demonstrating that we need different measures for the flexibility of inorganic ligands to #HA, TPSA, #HD and #rotatable.

Finally, the proposed analysis is also limited to the adopted comparison algorithm. The proposed method uses atomic coordinate comparison, which compared to shape comparison, has the problem of superposing binding sites composed of different numbers of atoms and atom types [11]. This echoes that this is an alternative analysis to other works since shape representation has its own problems in active sites without star-like shapes [11]. A model considering both the atomic details and shape characteristics is needed to this limitation. Another possible solution is to classify sites by their shapes and use the appropriate comparison algorithm accordingly.

## Conclusions

Knowing the flexibility of enzyme active sites is a crucial step of understanding the various binding mechanisms. There have been many studies examining this problem by selecting some flexible active sites and analyzing their evolutionary and structural conservation. This study, on the other hand, proposes an analysis framework to detect novel active sites with flexibility. The framework is composed of a new data collecting strategy, a local structure alignment tool and several physicochemical measures derived from the alignments. The experimental results show the applicability of combining the three components as well as its potential to identify flexible active sites. In general, the proposed analysis framework provides an alternative rather than a substitute of previous works. It is highly automated and robust so that more extensive studies are feasible in the future.

## Methods

### Pair-wise structure alignments of an active site group

In this study, an active site is represented by a group of PDB structures associated with annotated catalytic residues and the flexibility of an active site is obtained from the pair-wise structure alignments of its associated PDB structures. We define the local structure of an active site as the catalytic residues and those within 3Å of the catalytic residues, where the distance of two residues is the distance of their nearest heavy atoms. We define the local structure in this way because, although the catalytic residues play the most important role in ligand binding, the surrounding residues need to be conserved to provide a stable environment. A group of *n* local structures results in *n*(*n*–1)/2 alignments (*n* self-to-self alignments are not required).

This study adopts the CLoSA (Constraint-based Local Structure Alignment) algorithm to perform pair-wise structure alignment. It is an efficient local structure alignment tool with four constraints designed for active site comparison. The CLoSA algorithm is composed of three steps: cavity identification, structure comparison, and alignment scoring. In our implementation, this cavity identification step is disabled, since this study only aligns local structures that are already cavity-like by construction. The structure comparison step of CLoSA is based on the geometric hashing algorithm [31], where the alignment frames examined are defined by the two backbone bonds connected to the alpha carbon of each residue. This definition has been widely used when applying the geometric hashing algorithm to protein structure alignments [32,33]. In this step, two residues are regarded as successfully aligned if the distance between them is ≤5Å. Accordingly, the time complexity of the structure comparison step is *O*(*n*_1_*n*_2_(*n*_1_+*n*_2_)), where *n*_1_ and *n*_2_ denote the number of residues in the two compared local structures, respectively.

The most distinct feature of CLoSA is the inclusion of four constraints in the alignment scoring step. The first constraint ensures that ≥20% residues in the given structure are successfully aligned. The second constraint states that the RMSD (root mean square deviation) of the aligned alpha carbons must be ≤5Å. In our implementation, the third constraint of the opening direction is disabled to deal with active sites binding ligands of different orientations [34]. The fourth constraint ensures that SOC (sequence order conservation) ratio ≥0.37 and sRMSD (skew RMSD) ≤5Å of the aligned alpha carbons. SOC is the number of aligned residues having inconsistent sequence orders and sRMSD is an adjusted RMSD by the following equation:

,

where MAX_RMSD denotes the maximum distance between two aligned alpha carbons (5 Å in this study). The sRMSD represents a heuristic measure to penalize order mismatches by assigning them larger RMSD values. Finally, the alignments that passed all the constraints are ranked with the TM-score [35], a measure of the similarity of topologies of two proteins. TM-score is more sensitive than the RMSD of the aligned alpha carbons in accessing the quality of structure alignment.

## Competing interests

The authors declare that they have no competing interests.

## Authors' contributions

Author YCW designed the experiments and performed all calculations and analyses. DTHC designed the methodology and conceived the study. YFH aided in interpretation of the data and manuscript preparation. CWL participated in the data preparation. All authors have read and approved this manuscript.

## Supplementary Material

Additional file 1 **A collection of 58 active sites** This file includes the physicochemical properties of the 58 active site groups collected in this study. Click here for file
